# Understanding illness awareness in Alzheimer’s disease: a qualitative study with patient–caregiver dyads in Colombia

**DOI:** 10.3389/fnagi.2026.1742428

**Published:** 2026-02-04

**Authors:** Claudia Ramos, Claudia Madrigal, Jenny García, Margarita Giraldo, David Aguillón, Ivana Markova, Carlos Yepes

**Affiliations:** 1 Grupo de Neurociencias de Antioquia, Facultad de Medicina, Universidad de Antioquia, Medellín, Colombia; 2 Grupo Neuropsicología y Conducta, Facultad de Medicina, Universidad de Antioquia, Medellín, Colombia; 3 Facultad de Medicina, Universidad de Antioquia, Medellín, Colombia; 4 Hull York Medical School, University of Hull, Hull, United Kingdom; 5 Pablo Tobón Uribe Hospital, Medellín, Colombia

**Keywords:** Alzheimer’s disease, awareness, caregivers, cognitive dysfunction, dementia, grounded theory, social support

## Abstract

**Background:**

Diminished awareness of illness is a frequent neuropsychiatric symptom in people with Alzheimer’s disease. It has been linked to emotional and behavioral challenges such as depression, apathy, irritability, and poor adaptation to loss of function. Despite its clinical importance, little is known about how individuals and their caregivers understand and experience illness awareness, particularly in low- and middle-income countries.

**Objective:**

To explore the meaning of illness awareness and the factors associated with its impairment, as experienced and described by people living with Alzheimer’s disease and their caregivers in Medellín, Colombia.

**Methods:**

Semi-structured interviews were conducted with 20 individuals diagnosed with major neurocognitive disorder due to Alzheimer’s disease and 24 caregivers. A grounded theory approach guided data collection and analysis, allowing for an inductive exploration of shared meanings and contextual influences.

**Results:**

For these patient–caregiver dyads, being aware of illness meant recognizing cognitive, emotional, and behavioral changes linked to the neurodegenerative process. Awareness and its disruption were shaped by personal characteristics (such as personality and emotional resilience), prior life experiences, socioeconomic conditions, and the quality of social support. Caregivers who felt emotionally supported and well-informed about the disease were better equipped to support changes in awareness in the person with dementia without increasing distress.

**Conclusion:**

Understanding the cultural and interpersonal dimensions of illness awareness offers critical insights for the design of interventions that address absence of awareness with sensitivity and compassion. Recognizing how awareness is formed, preserved, or lost can inform more person-centered approaches to care.

## Introduction

Alzheimer’s disease (AD) is considered the most common dementia or Major Neurocognitive Disorder (MND) today, and is characterized by cognitive impairment, especially of memory and learning, and at least one other domain such as attention, executive function, language and social cognition (Patterson, 2018). However, 20–41% of people with mild to moderate AD (Turró-Garriga et al., 2013) and up to 81% of all patients with Major Neurocognitive Disorder due to Alzheimer’s Disease (MND due to AD) have issues in illness awareness, i.e., a decreased or loss of awareness of affective and behavioral changes and impairments in performing activities of daily living (Marková et al., 2014; Starkstein, 2014).

Although lack of awareness and impaired awareness are often referred to as anosognosia, it is important to clarify that these concepts are related but not identical. Anosognosia literally refers to a partial or total absence of awareness resulting from neurological causes (Markova, 2005). In contrast, impaired self-awareness is a broader and more complex construct that encompasses both neurological and non-neurological factors, including emotional and socio-cultural components. As highlighted by Prigatano et al. (2024), understanding lack of awareness in neurodegenerative diseases requires considering both neurobiological mechanisms and psychological processes such as denial.

Impairment of awareness has diverse neurophysiological bases and neuropsychological correlates. A 2022 systematic review of 36 neuroimaging studies identified mesial temporal involvement—particularly of the hippocampus—as well as hypometabolism in the anterior dorsal cingulate and parietotemporal cortices, and atrophy of the cerebellar vermis, left postcentral gyrus, and right fusiform gyrus as neural correlates of anosognosia (Leocadi et al., 2023). Individuals with cognitive impairment and anosognosia also exhibit a higher global cerebral amyloid burden compared with cognitively impaired individuals without anosognosia (Wang et al., 2024). In addition, event-related potential studies have shown deficits in error-monitoring processes, which impair the ability to detect errors and adjust behavior to support learning (Razafimahatratra et al., 2022). Finally, lack of self-awareness in individuals with mild cognitive impairment and dementia has been associated with neuropsychological deficits in cognitive domains such as speech, language, calculation, and short-term memory, even in the absence of aphasia (Coddaire et al., 2025).

Impaired awareness is an important neuropsychiatric problem as it is associated with longer hours of care, resistance to supervision, poorer adherence to treatment, and increased use of health services (Turró-Garriga et al., 2013).

It is likely that devising an intervention with a focus on impaired awareness would contribute to the maintenance of some autonomy and independence of patients and to achieve good teamwork with caregivers, thus increasing their level of self-efficacy. The latter is an aspect closely related to the subjective feeling of overload and the perception of a better or worse quality of life in general (Cristancho-Lacroix et al., 2015).

Addressing the absence or impairment of awareness is a relevant issue that should be considered in non-pharmacological treatment programs for people with MND due to AD, particularly to improve and sustain patient adherence, even among those with limited or no disease awareness. In a rapid systematic search of interventions targeting people with dementia and impaired awareness, only two studies were identified that aimed to enhance awareness as a primary outcome, while five addressed awareness as a secondary outcome, mainly focusing on other domains such as cognition, coping, quality of life, and neuropsychiatric symptoms (Alexander et al., 2025).

The scarcity of interventions specifically designed for patients with MND and impaired awareness may be related to concerns about causing distress or suffering if awareness increases. Consistent with this view, absence or lack of awareness has been described as a psychological defense mechanism that helps individuals cope with the emotional pain associated with cognitive and functional losses (Trindade et al., 2023; Thorsen et al., 2020). Moreover, awareness of cognitive deficits has been associated with a lower perceived quality of life in individuals with mild cognitive impairment and dementia (Stites et al., 2017). These findings highlight the importance of incorporating the perspectives of potential users when designing therapeutic strategies that address impaired awareness, in order to identify approaches that do not exacerbate suffering.

Accordingly, the aim of the present study was to explore the meaning of impaired disease awareness among patients with mild to moderate MND due to AD and their caregivers, to identify factors associated with its presence, and to examine potential ways of addressing it through intervention.

## Materials and methods

A hermeneutic methodology was used, based on grounded theory (GT) techniques (Strauss and Corbin, 2002). This approach allowed the identification of constructs from the information provided by the participants, from which the theory emerged. The basis of GT is symbolic interactionism, a philosophical current originating in sociology, whose objective is to help understand how individuals define a phenomenon based on social interaction, in order to subsequently generate explanatory theories of this phenomenon (Crabtree and Miller, 1999). This theory uses inductive reasoning to collect information, carry out an exhaustive and systematic analysis and organize the results (Verdinelli and Scagnoli, 2013). To develop the manuscript, the consolidated criteria for reporting qualitative research (COREQ) were followed (Tong et al., 2007).

### Population

A non-probabilistic selection or sampling was made, by convenience, of persons with MND due to AD and at least one of their family members or caregivers, referred by professionals in general medicine, neurology, psychology or neuropsychology of the Grupo de Neurociencias de Antioquia, GNA, after they authorized to be contacted. The eligible population consisted of patients with a diagnosis of AD and who had a caregiver as a companion; both the caregiver and the patient had to be over 18 years of age and agree to be study participants. To effects of the research, the caregiver had to be informal: a non-professional—typically a family member or close companion—who assumed responsibility for providing ongoing care, support, and decision-making without formal training or financial remuneration; a caregiver could be primary (a person who spent most of the time with the patient) or secondary (a person who gave supplemental support to care delivered by others) (Organisation mondiale de la santé, 2012).

The only exclusion criterion was the presence of significant language difficulties that hindered understanding of the questions. Both patients and caregivers were contacted by telephone and the objectives of the study were explained to them. All persons contacted agreed to participate.

### Alzheimer’s disease diagnosis, cognitive status, and disease severity

Of the 20 patients, four were diagnosed by a multidisciplinary clinical team at GNA, composed of specialists in neurology, psychiatry, and neuropsychology. These four patients, who had early-onset autosomal dominant Alzheimer’s disease (AD in people under 65 years old), were subsequently referred to their health insurance providers to receive pharmacological and non-pharmacological support.

Seven patients were diagnosed at GNA by a neurologist with expertise in Alzheimer’s disease and other neurodegenerative conditions. After receiving the diagnosis, these participants were also referred to their health insurance providers for pharmacological and non-pharmacological support.

The remaining nine patients were diagnosed by a neurologist affiliated with their health insurance company and later attended GNA seeking guidance through its psychoeducational support groups.

All participants were at a mild stage of the disease at the time they received their initial diagnosis. The diagnosis was disclosed to both the patient and a relative (spouse, child, or other close family member, such as a sibling).

At the time of the interviews, each diagnosis had been confirmed by a neurologist or psychiatrist at GNA, in accordance with the National Institute on Aging–Alzheimer’s Association criteria (McKhann et al., 2011). Additionally, patients were assessed using the Mini-Mental State Examination (MMSE) (Folstein et al., 1975) and the Clinical Dementia Rating (CDR) scale (Morris, 1993) to evaluate cognitive status and determine disease severity.

### Data collection techniques, instruments, and procedures

Data collection was based on an interview script, which had three sections: (1) preamble to explain the research and objectives; (2) collection of sociodemographic data; and (3) questions related to the research topic.

Initially, patients and their caregivers were invited to attend the visit in person, guaranteeing them compensation for transportation expenses. However, those who could not travel to the GNA facilities because they lived outside Medellín, had physical limitations or because of work-related occupations, were offered a virtual interview by video call. In this case, the patient was first interviewed independently followed by the caregiver who was then also interviewed independently. When face-to-face interviews were conducted, the patient was interviewed first and then the caregiver or vice versa. Therefore, all the participants were interviewed individually.

At the time of the interview, only the mental health professional and the patients or their caregivers were present. If any of the questions generated any type of emotional discomfort, this was managed by the intervention of the researcher in charge of the interview. The interviews were recorded using the Google Meet and Zoom platforms, the cell phone application *Easy Voice Recorder,* version 2.8.8, and the Sony ICD-PX240 digital recorder.

A pilot test of the interview protocol was carried out with a patient with MND due to AD and his caregiver. The pilot test allowed the necessary adjustments to be made to the questions asked. Each of the interviews was transcribed verbatim.

The study was made as robust as possible by: (1) allowing sufficient time to carry out the research, between two and three years; (2) ensuring a broad clinical and theoretical experience across the team, for which we had a male physician, master in public health and doctor in clinical epidemiology, with more than 20 years of experience in hermeneutic research and who served as a methodological expert and two female psychiatrists, of whom one had experience in qualitative research and the other had training and experience in the evaluation and management of patients with neurodegenerative diseases; and (3) using the detailed literature review to construct the material and methodology involved in the study (Wang et al., 2024; Alexander et al., 2025).

### Analysis plan

For the transcription and subsequent verification of the interviews, alphanumeric sequential coding was done for each interview, in order to safeguard the confidentiality of the data. For the analysis of the content of the interviews, the constant comparative method was used, that is, the information was collected, coded and analyzed systematically. The researchers met for six consecutive dates in order to standardize the coding process through the joint analysis of interviews 1 and 5. Three types of coding were used: open, axial and selective (Strauss and Corbin, 2002).

Both voices, that of patients with MND due to AD and that of caregivers, were allowed to converge in the determination of joint categories, given the need to identify convergences and divergences to be taken into account when thinking about the most appropriate way to address the issue of absence of awareness through an intervention with Information and Communication Technologies, ICTs, which would be applied to caregivers and patients together and not separately.

During data analysis we looked for both code saturation and meaning (Hennink et al., 2017). Code saturation, i.e., when categories other than those already identified ceased to emerge, was reached in the assessment of the fourth dyad (interviews No. 7 and 8). However, because the theme of *self-awareness* emerged during interview 13 and a better exploration of the psychological and social factors related to illness awareness and the strategies used by participants to lessen the negative consequences of absence of awareness was required, 13 more patients and 17 more caregivers were interviewed in order to reach meaning saturation as well.

RStudio version 2023.12.1 Build 402 was used to analyze the sociodemographic data. Microsoft Word version 16.54 was used to record the transcription of the interviews and to do the open coding; and Microsoft Excel version 16.54 was used for axial and selective coding. Artificial intelligence was not used for interview transcription or data analysis.

### Ethical considerations

Both patients and their caregivers agreed to participate and signed the informed consent and informed assent forms, respectively. According to resolution 8,430 of 1993 establishing the scientific, technical and administrative standards for health research in Colombia (Ministerio de Salud, 1993), this was a minimal risk investigation. The standards of the Declaration of Helsinki were followed, and the research protocol of this study was approved by the Bioethics Committee of the Faculty of Medicine of the University of Antioquia, minutes No. 27 of March 31, 2022 and No. 47 of April 27, 2023.

## Results

Twenty patients with MND due to AD and 24 caregivers participated. Of the patients interviewed, 16 had one caregiver and four had two caregivers who participated in the research. This occurred because additional caregivers, with different but complementary roles to those of the caregiver initially approached, also wanted to participate in the evaluation. Because they met the selection criteria, they were allowed to participate, although the interviews were conducted at different times than the patients and the data were processed separately. Among the participants with MND due to AD, 12 (60%) were female, with a mean age of 69.7 (*SD* 12.3) years. The vast majority (75%) of affected participants had late-onset MND due to AD (Table 1). Of the 24 caregivers, 17 (70.8%) were female. Half of the participants with MND had low socioeconomic levels (Table 2).

**Table 1:** Cognitive status of participants.

Participant with AD	MMSE	CDR	Type of Alzheimer’s disease^*^
1	26	1	Early
2	27	1	Early
3	22	1	Late
4	23	1	Early
5	23	1	Late
6	21	2	Late
7	20	1	Early
8	14	1	Late
9	15	2	Late
10	27	1	Late
11	15	2	Early
12	22	0.5	Late
13	20	1	Late
14	17	1	Late
15	23	1	Late
16	22	1	Late
17	18	1	Late
18	20	0.5	Late
19	22	2	Late
20	22	2	Late

MMSE, Mini-Mental State Examination; CDR, Clinical Dementia Rating Scale.

* Type of major neurocognitive disorder due to Alzheimer’s disease, according to age-at-onset: Early, in people under 65 years old; Late, in people who were 65 and older.

**Table 2:** Demographic characteristics of persons with Alzheimer’s disease and their caregivers.

	Patient with AD	Caregiver
*N*	20	24
Age (mean (SD))	69.65 (12.27)	52.75 (12.64)
Sex = female (%)	12 (60.0)	17 (70.8)
Type of AE = late (%)	15 (75.0)	N/A
Place of birth	n (%)	n (%)
Antioquia	13 (65.0)	18 (75.0)
Caldas	1 (5.0)	N/A
Chocó	1 (5.0)	N/A
Magdalena	N/A	1 (4.2)
Meta	N/A	1 (4.2)
Norte de Santander	2 (10.0)	1 (4.2)
Santander	1 (5.0)	1 (4.2)
Tolima	1 (5.0)	1 (4.2)
Cauca Valley	1 (5.0)	1 (4.2)
Residence = urban (%)	18 (90.0)	22 (91.7)
Marital status	n (%)	n (%)
Married	7 (35.0)	12 (50.0)
Separated	N/A	2 (8.3)
Widowed	6 (30.0)	1 (4.2)
Single	4 (20.0)	4 (16.7)
Unmarried	3 (15.0)	5 (20.8)
Schooling (mean (SD))	11.90 (3.43)	13.83 (3.14)
Socioeconomic stratum	n (%)	n (%)
Low Low	1 (5.0)	1 (4.2)
Low	3 (15.0)	3 (12.5)
Medium low	6 (30.0)	6 (25.0)
Medium	4 (20.0)	6 (25.0)
Upper middle	2 (10.0)	2 (8.3)
High	4 (20.0)	6 (25.0)
Relationship with the patient with AD (%)		
Partner	N/A	8 (33.3)
Child		12 (50.0)
Sibling		2 (8.3)
Other*		2 (8.3)

*Occupation in the affected persons refers to what they were doing before they retired or developed the disease.

AD, Alzheimer’s disease; N/A, not applicable; SD, standard deviation.

A total of 2,928 codes were identified, followed by 42 descriptive categories. Then 109 properties or attributes and seven phenomena or analytical categories were identified. Some examples of codes, descriptive categories and their respective properties or attributes can be reviewed in Table 3.

**Table 3:** Examples of codes grouped into different dimensions, descriptive categories, and their respective properties or attributes.

Code	Descriptive category	Property or attribute
E1P2: telling that her husband (the patient) is still very independent, so in the morning hours he turns around and tries to do a lot of things at home.	Autonomy	Some caregivers consider that autonomy is the ability to perform self-care activities by oneself or activities that are useful to others, which generates a sense of control in the person performing them, can be maintained through activities such as physical exercise, and is relevant for the person diagnosed with Alzheimer’s disease.
E2P22: say that she likes that her husband still manages money well (caregiver).		
E5P33: report that she is not allowed to go out alone because of falls (patient)		
E6P1: acknowledge that they do everything to her because of the disability she has		
E2P19: report that the person she cares for has recently become much more irritable (caregiver).	Caregiving burden	For some caregivers, the work of caregiving is exhausting due to the functional dependence and psychological and behavioral symptoms of the patients, working outside the home or caring for two or more people at the same time; they say that the overload makes them impatient, they feel guilty for the actions derived from their impatience and they would like to control their emotions better, so that emotions do not manage them and they can provide good care to their loved ones.
E2P21: feel that her husband’s personality makes it more difficult for her to suggest things to him regarding his well-being and care (caregiver).		
E4P1: find her experience of caring for a person with Alzheimer’s disease very draining, she pauses and is emotionally affected (caregiver).		
E7P2: feeling that caring for a patient is very horrible when she stops bathing for a long time (caregiver)		
E213: consider it problematic for people not to be aware of the disease process they may be having (patient)	Awareness of illness	Some patients and caregivers consider that being aware of having a disease such as Alzheimer’s facilitates the acceptance of support from both health professionals and their caregivers, family and friends, and may contribute to the healing process, should any curative treatment be developed in the future.
E2P13: consider that it is preferable to be aware of what is happening (patient referring to illness)		
E5P31: accepting the disease facilitates the search for and solution of the problem, even if it is partial (patient).		
E7P3: not agreeing to hide the diagnosis of dementia from her sister, because she had to start processing the pension, among other things, and her sister was going to ask her why it was necessary to do those errands (caregiver).		

For both people with MND due to AD and their caregivers, illness awareness is awareness of changes related to the process of cognitive decline, such as memory loss, the type and severity of functional impairment, as well as certain psychological and behavioral symptoms: depression, anxiety, irritability, delusions or erroneous beliefs, apathy, sleep disturbances, etc.

Some participants with cognitive impairment acknowledged their difficulties in remembering recent events, and a few showed that they had information about the causes and evolution of AD and had even participated in clinical trials with potentially useful drugs to slow down the deterioration. Several of those affected also expressed concern about the impact of their clinical condition at the family level. However, other participants were unaware of their diagnosis or of the functional impact and neuropsychiatric syndromes suffered at the time of the interview, as well as the implications that the progression of deterioration has for their future and that of their families.

Although caregivers do not consider awareness of the diagnosis essential and may fear its negative consequences, they wish their loved ones would accept support in daily activities (e.g., managing finances, driving, going out, cooking), develop awareness of behavioral problems, and adhere to treatments for diseases other than AD.

All the above was synthesized into a basic social process, the components of which are described below.

### Moving through the disease, in company, while saying goodbye to life control

**Figure 1:**
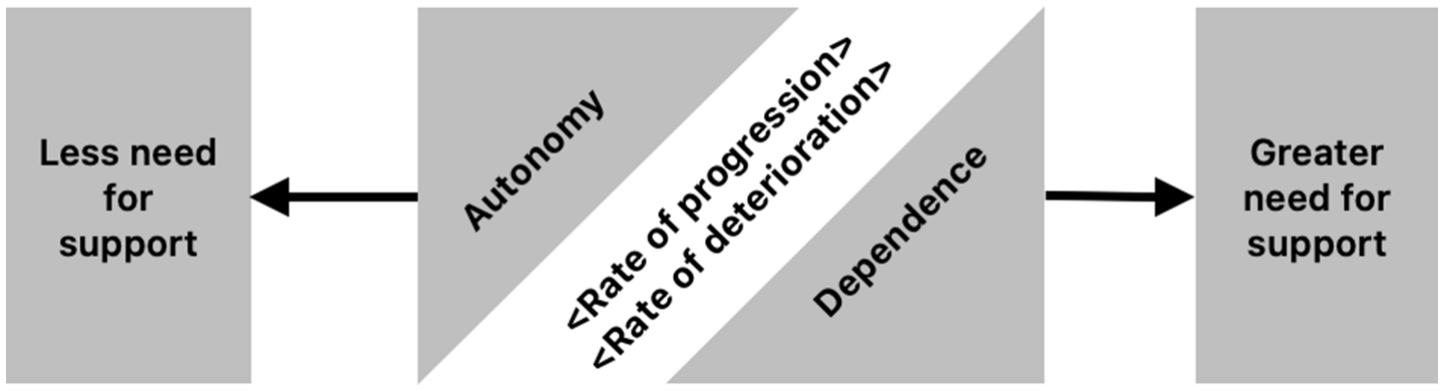
Struggling opposites in the patient with MND due to AD: autonomy versus dependence.

There are two phenomena, both significantly complex and going hand in hand in dialectical relationship, namely autonomy and dependence (Figure 1). At the onset of AD, there is usually some evidence of autonomy, which decreases over time, increasing the level of dependence. However, we speak of a dialectical relationship due to its complex non-linear dynamics, because it cannot be concluded that one simply results from the decrease or increase of the other.

This tense relationship between autonomy and dependence is related to the premorbid personality and is mediated by the evolution of the disorder, which does not behave in a linear fashion either. Such tension plays a leading role in the complex relationship between the patient with MND due to AD and his or her caregivers, family and friends, thus becoming one of the most relevant modulators of their interaction: being dependent for certain household tasks due to attentional complaints (dependence), yet claiming the right to go out unaccompanied (autonomy); wanting their loved one to feel useful (autonomy) but being aware of the need for supervision due to recurrent and possibly dangerous forgetfulness during tasks as cooking and ironing (dependence); allowing the patient the choice of clothes (autonomy), but helping him/her to get dressed (dependence).

*"(One needs a little bit of) Yes, a little bit of freedom in quotation marks, so yes, when I go out 'oh, don't go, so and so' and I 'oh and so', but as I tell you doctor, as far as I can I move because there will come a time when I won't, that's what happened with my siblings"* (Male patient, Interview 1a).

*“I don’t like being bothered, and she (my wife) gets angry very easily over nothing. So I take a taxi to avoid making things more complicated, and I go out, walk around the neighborhood, or go downtown”* (Male patient, Interview 4a).

*“She (the patient) was very street-oriented, very independent, so if she has awareness of herself, then it becomes very difficult”* (Female caregiver, Interview 3b).

### Illness awareness and self-awareness as bridges to connect with others and self

Illness awareness also goes from a higher to a lower level gradually and according to the participants depends, among other things, on three factors: (1) premorbid personality; (2) mood, which may fluctuate throughout the disease process; and (3) sociocultural factors such as schooling and purchasing power. Absence of illness awareness could be aggravated by a sense of distress at knowing that one is ill or recognizing the illness in others who are emotionally important to the patient.

*"I think the mere fact that you know you have this incurable disease is going to make you sicker"* (Male patient, Interview 1a).

*“Being aware that one is ill means pain, sadness, and anguish”* (male caregiver, Interview 6b).

*"I think that being aware of it is absolutely difficult and for example my mother… I would not want her to know because she lived through it with my aunts and she dreads it and she tells me 'oh no, anything but that for me'; she dreads it"* (Female caregiver, Interview 12b).

Religious beliefs may both reinforce absence of awareness—when distress is viewed as unnecessary or ungrateful—and protect illness awareness when suffering is framed as God-given strength to face daily challenges.

*"(On what it represents to be aware of an illness) …One is not given what one cannot, so if God gave it to me, besides, I am with their affection (daughters), with their support, with their friendship, tell me what else I ask from God"* (Female patient, Interview 12a).

Although initially linked to anger or sadness, illness awareness in patients with strong family support is no longer associated with negative emotionality, as they feel reassured to discuss and address the challenges of the disorder with others.

*“My husband would immediately refute me and become angry, saying things like, ‘Of course, I am useless, I am no longer useful.’ I would reassure him and calm him down, and his anger would subside. Now there is greater acceptance—of the disease and of me—which has created a very harmonious relationship between us”* (Female caregiver, Interview 16b).

In any case, a change in the awareness of the disease is expected as the disorder progresses, which does not seem to occur with self-awareness, which resists disappearing, although the deterioration continues.

*“Despite the disease, they still know who they are. It is like the soul or the essence of their being, which is never lost, even in very advanced dementia.”* (Female caregiver, Interview 7b).

Awareness of the disease becomes important to the extent that it conditions the relationship with family and friends; these, in turn, according to the characteristics of the interaction, will provide only accompaniment, only care, or the conjunction of these two types of relationship: an optimal or close social support.

### From accompaniment to care to close social support

**Figure 2:**
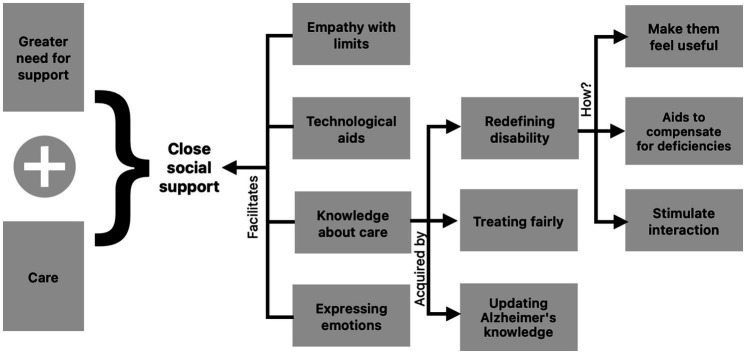
Close social support: the result of combining companionship and care.

The bond established with family and friends derives, on the one hand, from the tension between autonomy and dependence, a struggle in which dependence ends up winning, which eventually leads to a greater need for accompaniment. However, family and friends can accompany a person with dementia without necessarily implying care. In turn, it is possible to care for the person with MND due to AD, without the person receiving care feeling accompanied. But an intersection between accompaniment and care can be evident: optimal social support, labeled here as “close social support” (Figure 2).

Close social support emerges from the combination of companionship and care grounded in the empathy and compassion of caregivers or family members toward their ill loved one. Beyond the mere acceptance of caregiving responsibility, it requires perceiving the other as a suffering human being and being able to place oneself in their position in order to provide affection, reassurance, and a sense of calm and security that supports their physical, emotional, and spiritual well-being.

*“They—my siblings, including my brothers—talk to me very affectionately. They try to make sure I don’t feel bad. Instead, they think about working with me, and they gently remind me of things so I don’t forget, so that I can make calls or do what I need to do. Do you understand?”* (Female patient, Interview 7a).

*“People often tell me, ‘I couldn’t do it, you must have a lot of patience.’ But I feel it is not about wanting or not wanting to care; it is about putting yourself in the other person’s place and feeling compassion for them”* (Female caregiver, Interview 9b).

Both caregivers and professionals in the area should work with and for patients with AD, trying to team up with them, which will help to avoid the feeling of compassion fatigue, a situation derived from excessive empathy that is potentially preventable if there is cooperation among all the actors involved in the disease process.

*"(I) ask for that (help with medications from his wife) because I go with her wherever she needs to go."* (Male patient, Interview 16a, referring to the relationship with his partner, which managed to become a kind of mutualism: she helps him with the medication, and he accompanies her on her errands).

*“I also say that I haven’t searched very much because, honestly, I don’t have much time. But when you come to this memory clinic and ask any of the doctors who treat him, they immediately guide you toward what could be considered the ideal way to care for them. Of course, each human being is very different, but there has been a lot of guidance”* (Female caregiver, Interview 1b).

The “close social support” is also configured and enhanced through technological aids that allow respectful monitoring of the loved one with the condition, learning acquired about caregiving and the expression of emotions by the caregivers.

### Re-defining disability through technology and knowledge about the disease

Disability can be understood as a different way of making sense of the one who has a disease such as MND due to AD, in order to be able to contribute to the satisfaction of needs related to cognitive deficit and to the emotional, behavioral and functional difficulties of the patient.

*“Disability is not only physical, but also cognitive. A person who cannot be left alone is already living with a disability: someone who cannot go out on their own, becomes spatially disoriented even within the home, cannot light the stove or perform basic tasks, and requires 24-hour accompaniment”* (Female caregiver, Interview 8c).

By redefining AD and seeing it more as a type of disability, it is possible to search for ways to enhance the knowledge acquired about brain health, Alzheimer’s disease and caregiving, perhaps through technological tools, with the aim of providing good care and transmitting to others the feeling of close social support that makes the pain of knowing they are unwell more bearable.

*“Yes, we like cell phones and computers, because they keep us connected to the world and up to date on these kinds of illnesses, which is very, very important”* (Female caregiver, Interview 6c).

*“When I was caring for my mother, I used to listen a lot to* Radio Bolivariana*. They broadcast many health and psychology programs; it was a very good station. I also went to the library to read. Whenever I had some time, I would slip away and read a lot about medicine and nursing, because she was already very ill… things like changing diapers and all that. Now, to care for my sister, I keep myself updated through my phone, researching on YouTube”* (Female caregiver, Interview 7b).

*"(Technology) is an excellent thing because my dad doesn't have patience, right, so… suddenly, so that we don't have to go there because of the distance, but that together we can connect to a meeting, and they can advise us"* (Male caregiver, Interview 5b).

*“All of this technology has helped us a lot, because sometimes he becomes very stubborn. No matter how much I insist, he decides to leave. I stay at home saying, ‘My love, don’t go,’ and he replies, ‘Do you think I’m not capable? Of course, I am.’ And he leaves. So, to avoid too much conflict, I try to distract him, so he forgets about it and calms down. But when that doesn’t work, God bless the bracelet and the phone”* (Female caregiver, Interview 1b).

*“I used to talk with all my friends on my cell phone. My teacher would call me to practice a song I had learned, and I would send it to him on WhatsApp. That’s what I used to do”* (Female patient, Interview 5a).

### Expressing emotions to comfort the soul and care, without suffering the burdens

When caring for a person with MND due to AD, negative emotions are usually experienced, such as anguish or worry about the patient’s ideation of death or suicide.

Moreover, caregivers can feel hopelessness when thinking that with pathologies such as AD “there is nothing to do,” and burden when faced with symptoms such as apathy. Apathy is a neuropsychiatric symptom that hinders the patients’ response to the efforts of their loved ones to slow down the deterioration.

*“She used to enjoy reading, but she stopped doing crossword puzzles, stopped doing Sudoku, stopped doing things altogether. Reading was the only thing left—she would read El Tiempo, her daily newspaper—and then she developed a fear of it”* (Female caregiver, Interview 3b).

*"For me, for me personally the hardest thing is when the patient loses the desire for everything"* (Female caregiver, Interview 16b).

**Figure 3:**
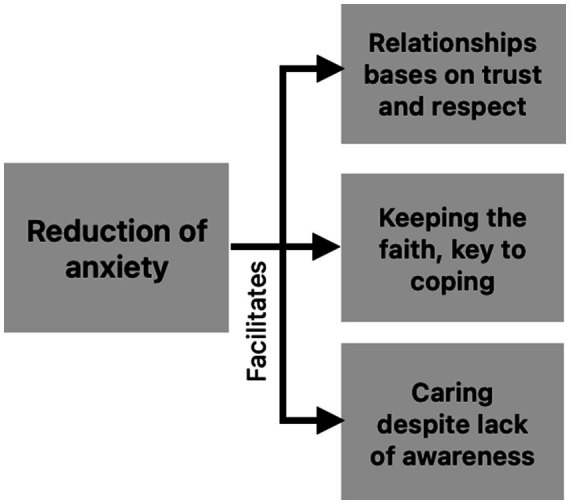
Benefits of reduced distress associated with the Alzheimer’s experience.

These emotions may emerge at any time and expressing them without fear of judgment can support better family caregiving. Caregivers’ adaptive processes aim to reduce the anguish associated with an illness still perceived as incurable, which in turn helps restore and strengthen the patient–caregiver relationship. This relief also sustains faith as a coping resource, allowing both to care and be cared for with greater peace of mind, even when the patient is unaware of the diagnosis and the caregiver avoids naming “Alzheimer’s disease” (Figure 3).

*“With the wisdom that the Lord has given me, I want to become a better person every day—but from within my heart, not outwardly, because outside I find nothing, doctor”* (Female caregiver, Interview 9b).

*“Without faith, one is nothing. Faith in the human being is what matters—if you do not have faith, you may have all the money in the world, you may have everything, but without faith you will not do well anywhere”* (Male patient, Interview 2a).

*“For God, everything is possible, and one must accept whatever comes with love”* (Female patient, Interview 5a).

### Absence of awareness: a complex process

**Figure 4:**
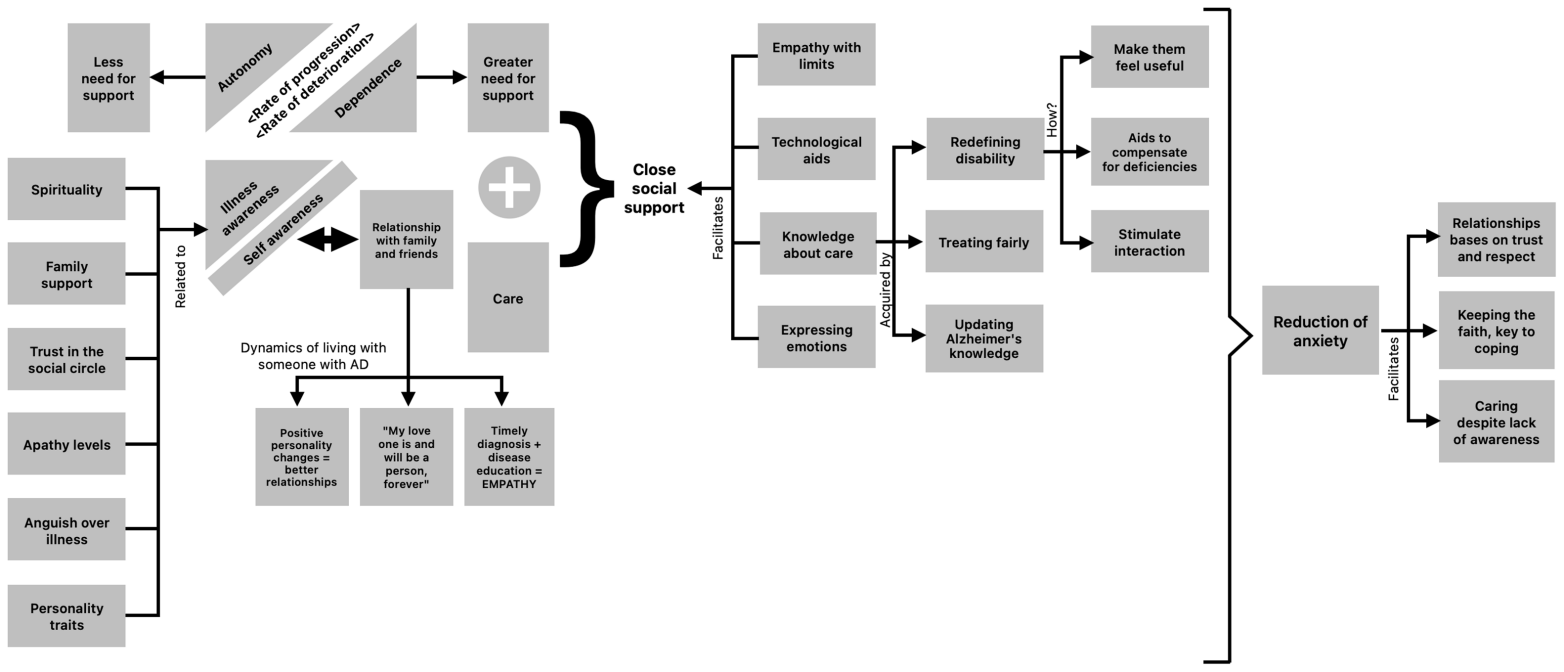
Complexity of the phenomenon of illness awareness in people with major neurocognitive disorder due to Alzheimer’s disease.

The scheme shown below (Figure 4) is based on the analytical categories: “autonomy,” “accompaniment,” “dependence,” “awareness of illness,” “self-awareness,” “coping” and “close social support.” It is thus possible to appreciate the confluence of all the categories described in a basic social process that allows us to understand how complex this whole phenomenon is. It is presumed that efforts to understand this process will contribute to effectively intervene in the complicated situation suffered by the dyads.

## Discussion

People affected by AD and their relatives and caregivers perceive illness consciousness not only as the result of adequate brain functioning, but also depending on the patient’s personality, mood, and certain sociocultural factors, which facilitate or hinder the process of acceptance of the neurocognitive disorder. Consciousness itself is a complex phenomenon, given that the structural sufficiency of the brain is not enough to possess this faculty, requiring in turn social interaction as a fundamental driving force in the development of one of the abilities to which the capacity to be conscious is attributed: language. Language contributes to the capacity to think, and with thought arises consciousness. Therefore, consciousness is the product of the life of the human being in society, constituting a social phenomenon (Yajot, 1973).

The loss of awareness of life in general and of the disease in particular, occurs gradually as the cognitive impairment progresses, which leads to a struggle between different opposites or mutually exclusive phenomena: autonomy versus dependence, less need for support versus greater need for support, ability, competence or capacity versus disability. At any moment of the clash between any of these opposites, a contradiction will always arise, given the “aspiration” of each to have a dominant significance in the patient’s life.

Far from signaling a complete lack of awareness in the MND due to AD, the struggle of opposites evidences an ambivalent illness awareness, oscillating between avoidance, exploration and understanding of the disorder, according to the results of a study published by Lishman et al. (2016) on the assimilation of the diagnosis of dementia. The researchers analyzed the interviews in light of the construct “community of voices,” which speaks of the *self* not as a unit, but as a multiple changing, context-dependent *self* (Lishman et al., 2016; McNamee and Gergen, 1992; Hermans et al., 1992).

When a traumatic experience threatens the identity of the affected person, a conflict is established between two opposites: the *Dominant Voice*, which resists change and encourages the defense of autonomy and independence; and the *Problematic Voice*, which carries uncertainty or sadness, that voice that warns of the journey toward dependence, given the gradual and probably irreversible loss of cognition (Lishman et al., 2016). With the passage of time, it seems that the affected person oscillates between these voices, sometimes behaving irrationally, looking as poor aware of their illness by defending the idea of not needing the other or requiring adjustments to survive, but at other times having a more sensible attitude, accepting the accompaniment of his or her support network. In the participants of our research this ambivalence or struggle between the *Dominant* and *Problematic* voices was also evident; this dialectic relationship is central to the therapeutic process, since the conversation of these voices favors the process of assimilation, with the changes that are required to be adopted in order to adjust to the needs related to the diagnosis.

This dialectical relationship between autonomy and dependence, which is reflected in fluctuations in levels of self-awareness and illness awareness, is shaped by premorbid personality and disease severity. However, the socio-cultural context of people living with Alzheimer’s disease appears to play a critical role in processes of acceptance and adjustment. In an article published by Thorsen et al. (2020), the authors describe how 10 individuals with MND, most of whom had early-onset AD, gradually changed their perceptions of their illness experience. Notably, one participant adopted a more positive attitude toward her condition after moving into a residential building shared with people of different ages and disabilities. Other participants also experienced improvements in mood after relocating to residential care settings, where they engaged more frequently with fellow residents.

In another study conducted by Trindade et al. (2023), which interviewed nine users of a day-care center diagnosed with mild to moderate MND, social relationships were shown to influence not only psychosocial well-being but also individuals’ willingness to express self-awareness and awareness of illness. Specifically, participants were more willing to acknowledge difficulties related to their brain health when interacting with family members and friends, compared with interactions with unfamiliar or distant individuals. Similar findings emerged in our study: feelings of familiarity and trust appeared to foster the emergence and maintenance of a certain level of illness awareness.

The characteristics of interpersonal relationships, especially those between patients and their support network, are critical to mitigate the severity and impact of absence of awareness, and to always treat those with AD as individuals. Lack of awareness and the presence of other frequent and burdensome neuropsychiatric symptoms that coexist with unawareness as apathy (Gallingani et al., 2025; Elnemais Fawzy et al., 2025), significantly affects how patients interact with their environment. Nevertheless, also family, friends and caregivers modify the way the adjustment process unfolds. Through empathic treatment and efforts to communicate despite the gradual deterioration of cognition, the affected person will see him or herself as valuable, which will help him or her feel secure as he or she adapts to his or her new disease condition and the evolution of the disorder (Thorsen et al., 2020).

Regarding the requirements to maintain an empathic treatment, it is essential that caregivers are able to express their emotionality regarding the diagnosis and clinical evolution of patients, in addition to learning about the disease, for which they can use the technology (Nissen et al., 2018). Talking about one’s own emotions and learning about AD would contribute to a moment of care that Carmen de la Cuesta calls “collaborative,” which arises when the person affected by the disease is in a state of “collaboration” (de la Cuesta Benjumea, 2004), which arises when the affected person actively contributes to his/her care, making it a joint action, since the individual takes care of him/herself and accepts the care of his/her companion.

Caregivers, family members, and companions of people with acquired brain injury may fluctuate between diametrically opposed positions regarding their loved ones’ lack of awareness of illness, as shown in a study published by Fidder et al. (2025). These positions include viewing impaired awareness as a symptom of the disease versus interpreting it as intentional behavior, as well as attempting to avoid conflict with the loved one versus choosing confrontation. In that study and in our research, more empathic and conciliatory perceptions and responses—such as learning about the loved one’s condition, understanding behavioral changes as part of the disorder, and avoiding confrontation by replacing it with collaborative efforts to support and maintain the patient’s autonomy—appeared to facilitate caregiving despite poor illness awareness.

An empathic attitude is important not only in the relationship between people with MND and their caregivers or family members, but also in interactions with the professionals responsible for diagnosis and clinical follow-up. In this study, participants showed varying levels and contents of illness awareness: some were more aware of their cognitive and functional difficulties, while others were more concerned about emotional aspects of their condition. Notably, patients and families who received care from professionals at a specialized dementia clinic appeared calmer and more reassured. Previous research by Stites et al. (2023) has shown an association between illness awareness and mental health decline in older adults with minor and major neurocognitive disorders. This relationship may be influenced by how the diagnosis is communicated, whether trusted companions are involved, and whether individual concerns about brain health are explored and addressed during clinical encounters (Stites et al., 2023; O’Brien et al., 2025).

Finally, in the present study, faith turned out to be one of the modulators of pain and distress associated with MND such as AD, a finding that has also been evidenced in other investigations where religion seems to be associated with positive feelings such as optimism, life purpose, generosity toward others and gratitude (Prestes, 2017). However, it would appear that religion and spirituality positively impact caregivers if their caregiving is meaningful to them or may affect them if they behave as guilt-generating by feeling bad while caring for their loved ones (Polson et al., 2024).

This study has some limitations. When the dyads were to be interviewed virtually, it was requested that the patient be alone in the space where he/she was to receive the video call and vice versa, that is, that the caregiver was not near the patient when it was his/her turn to be interviewed. However, it is not possible to guarantee that this condition was fully met and that there was no bias in the responses for fear of being heard by someone other than the researcher/evaluator. On the other hand, it is likely that there was heterogeneity in the depth of each of the descriptive and analytical categories. In addition, it would have been useful to measure the lack of awareness in MND due to AD, to know how people with varying degrees of absence of awareness viewed their clinical condition and concepts such as disease awareness, but this study did not contemplate it.

In conclusion, illness awareness is a fundamental aspect to consider in the development and implementation of treatment strategies for people with MND due to AD and their caregivers. Absence of awareness is not only explained by neurodegeneration, but also by critical aspects of personality, mood and certain socioeconomic and cultural characteristics. Such factors account for the dynamic nature of this phenomenon, which oscillates between the fear of losing autonomy and the acceptance of help from caregivers, family and friends in order to survive. This struggle within patients may be less painful for them if they perceive close social support, which can be fostered through a therapeutic strategy of teaching about AD, the causes and complications of poor disease awareness, practical advice on emotional management, and how to communicate empathetically, all potentially useful learning to mitigate the ravages of absence of awareness.

## Data Availability

The raw data supporting the conclusions of this article will be made available by the authors, without undue reservation.
